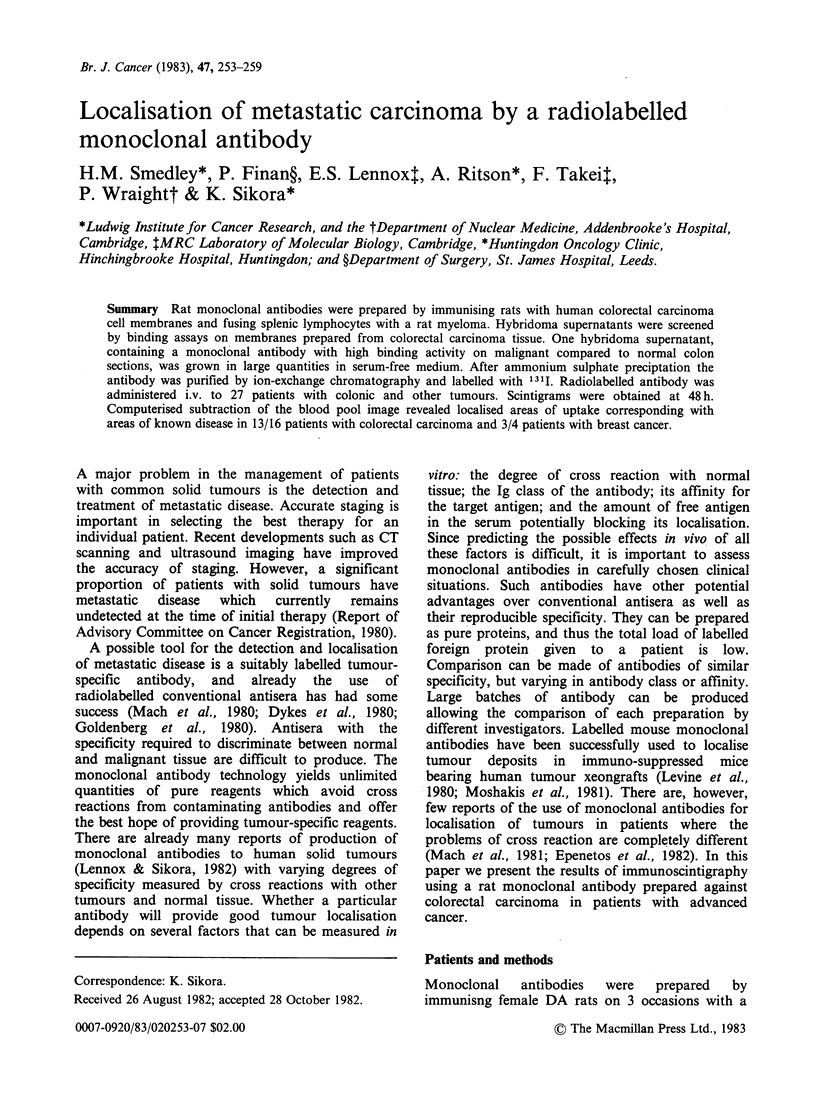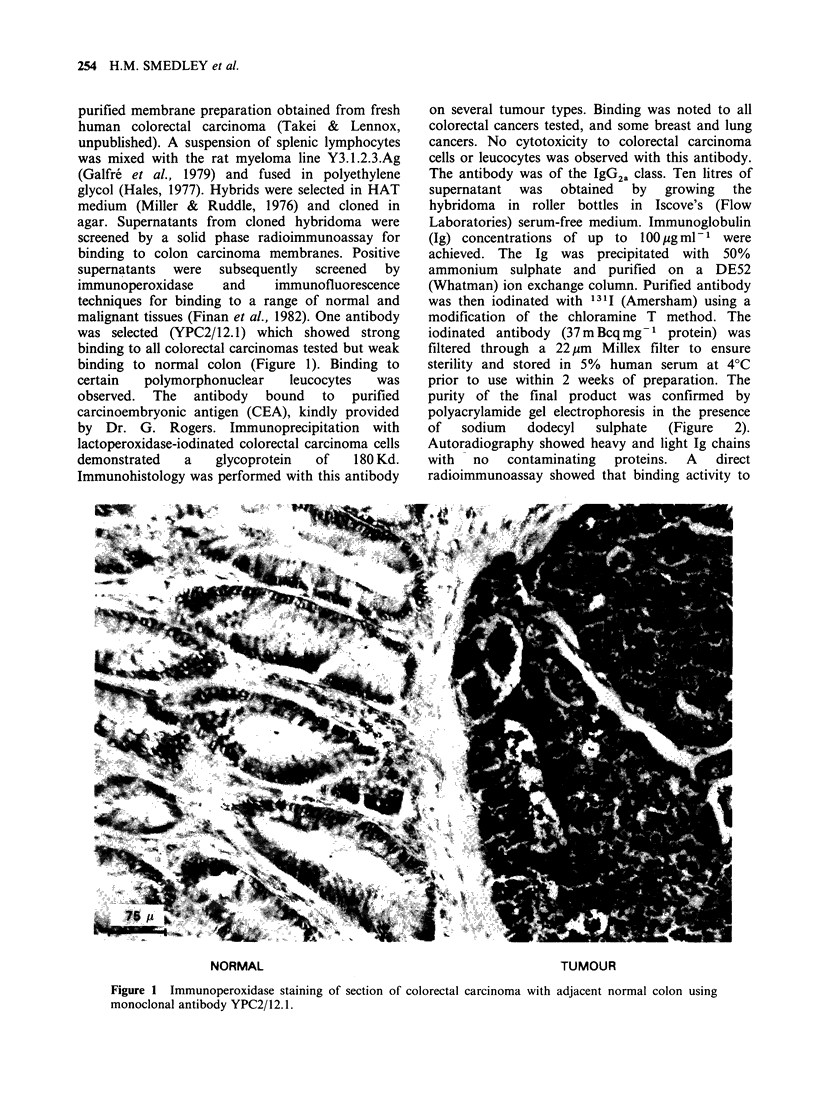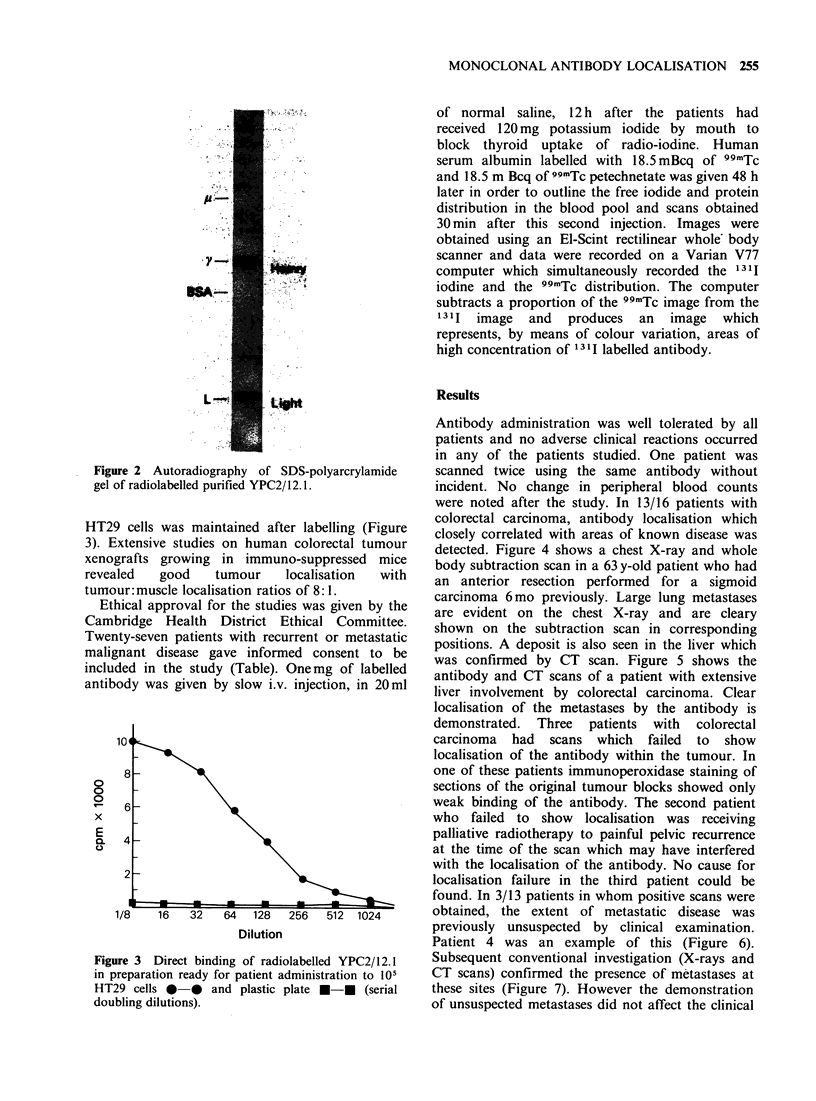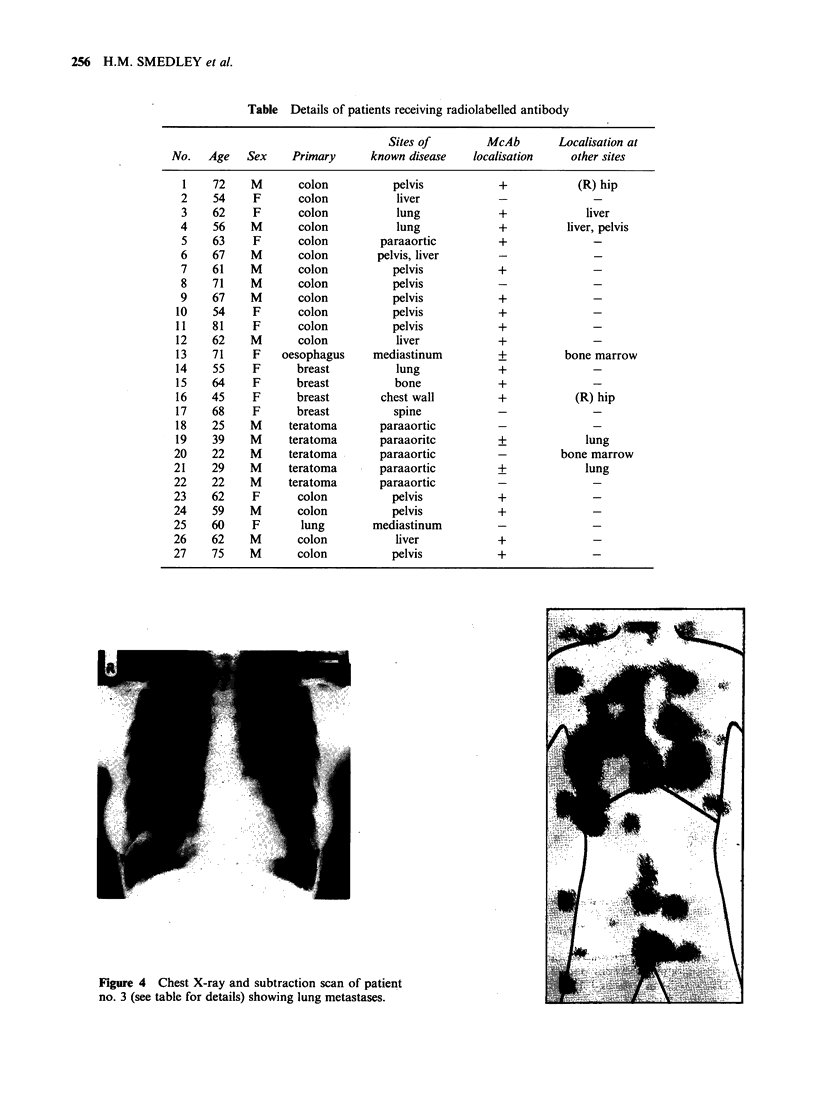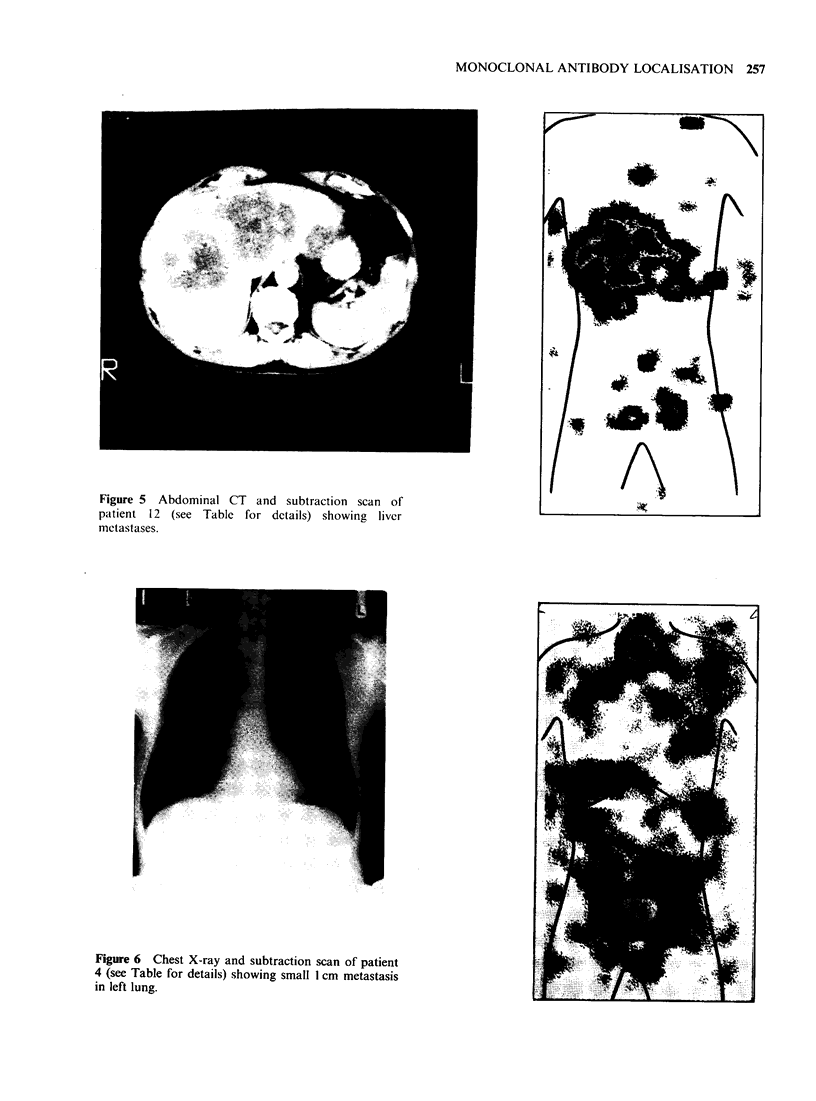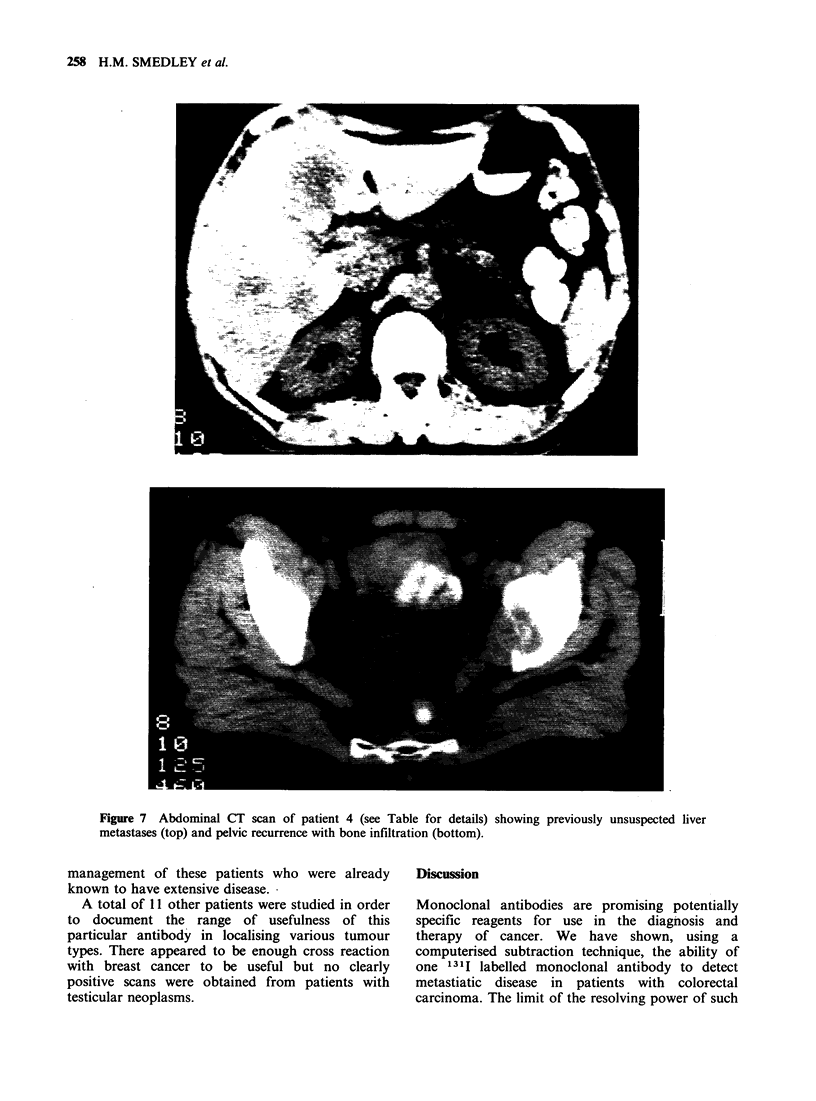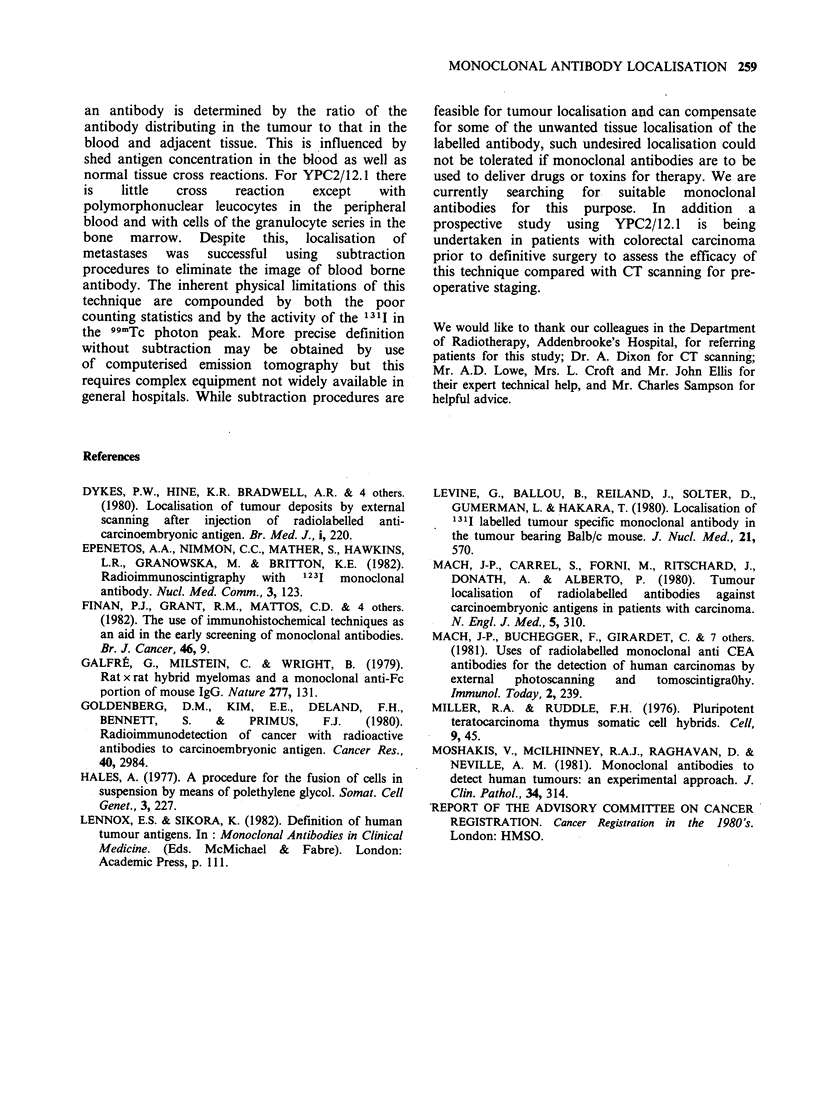# Localisation of metastatic carcinoma by a radiolabelled monoclonal antibody.

**DOI:** 10.1038/bjc.1983.33

**Published:** 1983-02

**Authors:** H. M. Smedley, P. Finan, E. S. Lennox, A. Ritson, F. Takei, P. Wraight, K. Sikora

## Abstract

**Images:**


*Br. J. Cancer* (1983), **47**, 253–259

### Localisation of metastatic carcinoma by a radiolabelled monoclonal antibody

H.M. Smedley*, P. Finan§, E.S. Lennox‡, A. Ritson*, F. Takei‡, P. Wraight† & K. Sikora*

**Ludwig Institute for Cancer Research, and the †Department of Nuclear Medicine, Addenbrooke's Hospital, Cambridge, ‡MRC Laboratory of Molecular Biology, Cambridge, *Huntingdon Oncology Clinic, Hinchingbrooke Hospital, Huntingdon; and §Department of Surgery, St. James Hospital, Leeds.*

**Summary** Rat monoclonal antibodies were prepared by immunising rats with human colorectal carcinoma cell membranes and fusing splenic lymphocytes with a rat myeloma. Hybridoma supernatants were screened by binding assays on membranes prepared from colorectal carcinoma tissue. One hybridoma supernatant, containing a monoclonal antibody with high binding activity on malignant compared to normal colon sections, was grown in large quantities in serum-free medium. After ammonium sulphate preciptation the antibody was purified by ion-exchange chromatography and labelled with $^{131}$I. Radiolabelled antibody was administered i.v. to 27 patients with colonic and other tumours. Scintigrams were obtained at 48 h. Computerised subtraction of the blood pool image revealed localised areas of uptake corresponding with areas of known disease in 13/16 patients with colorectal carcinoma and 3/4 patients with breast cancer.

A major problem in the management of patients with common solid tumours is the detection and treatment of metastatic disease. Accurate staging is important in selecting the best therapy for an individual patient. Recent developments such as CT scanning and ultrasound imaging have improved the accuracy of staging. However, a significant proportion of patients with solid tumours have metastatic disease which currently remains undetected at the time of initial therapy (Report of Advisory Committee on Cancer Registration, 1980).

A possible tool for the detection and localisation of metastatic disease is a suitably labelled tumour-specific antibody, and already the use of radiolabelled conventional antisera has had some success (Mach *et al.*, 1980; Dykes *et al.*, 1980; Goldenberg *et al.*, 1980). Antisera with the specificity required to discriminate between normal and malignant tissue are difficult to produce. The monoclonal antibody technology yields unlimited quantities of pure reagents which avoid cross reactions from contaminating antibodies and offer the best hope of providing tumour-specific reagents. There are already many reports of production of monoclonal antibodies to human solid tumours (Lennox & Sikora, 1982) with varying degrees of specificity measured by cross reactions with other tumours and normal tissue. Whether a particular antibody will provide good tumour localisation depends on several factors that can be measured *in vitro:* the degree of cross reaction with normal tissue; the Ig class of the antibody; its affinity for the target antigen; and the amount of free antigen in the serum potentially blocking its localisation. Since predicting the possible effects *in vivo* of all these factors is difficult, it is important to assess monoclonal antibodies in carefully chosen clinical situations. Such antibodies have other potential advantages over conventional antisera as well as their reproducible specificity. They can be prepared as pure proteins, and thus the total load of labelled foreign protein given to a patient is low. Comparison can be made of antibodies of similar specificity, but varying in antibody class or affinity. Large batches of antibody can be produced allowing the comparison of each preparation by different investigators. Labelled mouse monoclonal antibodies have been successfully used to localise tumour deposits in immuno-suppressed mice bearing human tumour xeongrafts (Levine *et al.*, 1980; Moshakis *et al.*, 1981). There are, however, few reports of the use of monoclonal antibodies for localisation of tumours in patients where the problems of cross reaction are completely different (Mach *et al.*, 1981; Epenetos *et al.*, 1982). In this paper we present the results of immunoscintigraphy using a rat monoclonal antibody prepared against colorectal carcinoma in patients with advanced cancer.

### Patients and methods

Monoclonal antibodies were prepared by immunisng female DA rats on 3 occasions with a

Correspondence: K. Sikora.

Received 26 August 1982; accepted 28 October 1982.

0007-0920/83/020253-07 $02.00

© The Macmillan Press Ltd., 1983

purified membrane preparation obtained from fresh human colorectal carcinoma (Takei & Lennox, unpublished). A suspension of splenic lymphocytes was mixed with the rat myeloma line Y3.1.2.3.Ag (Galfré *et al.*, 1979) and fused in polyethylene glycol (Hales, 1977). Hybrids were selected in HAT medium (Miller & Ruddle, 1976) and cloned in agar. Supernatants from cloned hybridoma were screened by a solid phase radioimmunoassay for binding to colon carcinoma membranes. Positive supernatants were subsequently screened by immunoperoxidase and immunofluorescence techniques for binding to a range of normal and malignant tissues (Finan *et al.*, 1982). One antibody was selected (YPC2/12.1) which showed strong binding to all colorectal carcinomas tested but weak binding to normal colon (Figure 1). Binding to certain polymorphonuclear leucocytes was observed. The antibody bound to purified carcinoembryonic antigen (CEA), kindly provided by Dr. G. Rogers. Immunoprecipitation with lactoperoxidase-iodinated colorectal carcinoma cells demonstrated a glycoprotein of 180 Kd. Immunohistology was performed with this antibody on several tumour types. Binding was noted to all colorectal cancers tested, and some breast and lung cancers. No cytotoxicity to colorectal carcinoma cells or leucocytes was observed with this antibody. The antibody was of the $IgG_{2a}$ class. Ten litres of supernatant was obtained by growing the hybridoma in roller bottles in Iscove's (Flow Laboratories) serum-free medium. Immunoglobulin (Ig) concentrations of up to $100\,\mu g\,ml^{-1}$ were achieved. The Ig was precipitated with 50% ammonium sulphate and purified on a DE52 (Whatman) ion exchange column. Purified antibody was then iodinated with $^{131}I$ (Amersham) using a modification of the chloramine T method. The iodinated antibody ($37\,m\,Bcq\,mg^{-1}$ protein) was filtered through a $22\,\mu m$ Millex filter to ensure sterility and stored in 5% human serum at 4°C prior to use within 2 weeks of preparation. The purity of the final product was confirmed by polyacrylamide gel electrophoresis in the presence of sodium dodecyl sulphate (Figure 2). Autoradiography showed heavy and light Ig chains with no contaminating proteins. A direct radioimmunoassay showed that binding activity to

**Figure 1** Immunoperoxidase staining of section of colorectal carcinoma with adjacent normal colon using monoclonal antibody YPC2/12.1.

**Figure 2** Autoradiography of SDS-polyarcrylamide gel of radiolabelled purified YPC2/12.1.

HT29 cells was maintained after labelling (Figure 3). Extensive studies on human colorectal tumour xenografts growing in immuno-suppressed mice revealed good tumour localisation with tumour:muscle localisation ratios of 8:1.

Ethical approval for the studies was given by the Cambridge Health District Ethical Committee. Twenty-seven patients with recurrent or metastatic malignant disease gave informed consent to be included in the study (Table). One mg of labelled antibody was given by slow i.v. injection, in 20 ml

**Figure 3** Direct binding of radiolabelled YPC2/12.1 in preparation ready for patient administration to $10^5$ HT29 cells ●—● and plastic plate ■—■ (serial doubling dilutions).

of normal saline, 12 h after the patients had received 120 mg potassium iodide by mouth to block thyroid uptake of radio-iodine. Human serum albumin labelled with 18.5 mBcq of $^{99m}$Tc and 18.5 m Bcq of $^{99m}$Tc petechnetate was given 48 h later in order to outline the free iodide and protein distribution in the blood pool and scans obtained 30 min after this second injection. Images were obtained using an El-Scint rectilinear whole body scanner and data were recorded on a Varian V77 computer which simultaneously recorded the $^{131}$I iodine and the $^{99m}$Tc distribution. The computer subtracts a proportion of the $^{99m}$Tc image from the $^{131}$I image and produces an image which represents, by means of colour variation, areas of high concentration of $^{131}$I labelled antibody.

### Results

Antibody administration was well tolerated by all patients and no adverse clinical reactions occurred in any of the patients studied. One patient was scanned twice using the same antibody without incident. No change in peripheral blood counts were noted after the study. In 13/16 patients with colorectal carcinoma, antibody localisation which closely correlated with areas of known disease was detected. Figure 4 shows a chest X-ray and whole body subtraction scan in a 63 y-old patient who had an anterior resection performed for a sigmoid carcinoma 6 mo previously. Large lung metastases are evident on the chest X-ray and are cleary shown on the subtraction scan in corresponding positions. A deposit is also seen in the liver which was confirmed by CT scan. Figure 5 shows the antibody and CT scans of a patient with extensive liver involvement by colorectal carcinoma. Clear localisation of the metastases by the antibody is demonstrated. Three patients with colorectal carcinoma had scans which failed to show localisation of the antibody within the tumour. In one of these patients immunoperoxidase staining of sections of the original tumour blocks showed only weak binding of the antibody. The second patient who failed to show localisation was receiving palliative radiotherapy to painful pelvic recurrence at the time of the scan which may have interfered with the localisation of the antibody. No cause for localisation failure in the third patient could be found. In 3/13 patients in whom positive scans were obtained, the extent of metastatic disease was previously unsuspected by clinical examination. Patient 4 was an example of this (Figure 6). Subsequent conventional investigation (X-rays and CT scans) confirmed the presence of mėtastases at these sites (Figure 7). However the demonstration of unsuspected metastases did not affect the clinical

**Table** Details of patients receiving radiolabelled antibody

| *No.* | *Age* | *Sex* | *Primary* | *Sites of known disease* | *McAb localisation* | *Localisation at other sites* |
|---|---|---|---|---|---|---|
| 1 | 72 | M | colon | pelvis | + | (R) hip |
| 2 | 54 | F | colon | liver | – | – |
| 3 | 62 | F | colon | lung | + | liver |
| 4 | 56 | M | colon | lung | + | liver, pelvis |
| 5 | 63 | F | colon | paraaortic | + | – |
| 6 | 67 | M | colon | pelvis, liver | – | – |
| 7 | 61 | M | colon | pelvis | + | – |
| 8 | 71 | M | colon | pelvis | – | – |
| 9 | 67 | M | colon | pelvis | + | – |
| 10 | 54 | F | colon | pelvis | + | – |
| 11 | 81 | F | colon | pelvis | + | – |
| 12 | 62 | M | colon | liver | + | – |
| 13 | 71 | F | oesophagus | mediastinum | ± | bone marrow |
| 14 | 55 | F | breast | lung | + | – |
| 15 | 64 | F | breast | bone | + | – |
| 16 | 45 | F | breast | chest wall | + | (R) hip |
| 17 | 68 | F | breast | spine | – | – |
| 18 | 25 | M | teratoma | paraaortic | – | – |
| 19 | 39 | M | teratoma | paraaoritc | ± | lung |
| 20 | 22 | M | teratoma | paraaortic | – | bone marrow |
| 21 | 29 | M | teratoma | paraaortic | ± | lung |
| 22 | 22 | M | teratoma | paraaortic | – | – |
| 23 | 62 | F | colon | pelvis | + | – |
| 24 | 59 | M | colon | pelvis | + | – |
| 25 | 60 | F | lung | mediastinum | – | – |
| 26 | 62 | M | colon | liver | + | – |
| 27 | 75 | M | colon | pelvis | + | – |

**Figure 4** Chest X-ray and subtraction scan of patient no. 3 (see table for details) showing lung metastases.

**Figure 5** Abdominal CT and subtraction scan of patient 12 (see Table for details) showing liver metastases.

**Figure 6** Chest X-ray and subtraction scan of patient 4 (see Table for details) showing small 1 cm metastasis in left lung.

**Figure 7** Abdominal CT scan of patient 4 (see Table for details) showing previously unsuspected liver metastases (top) and pelvic recurrence with bone infiltration (bottom).

management of these patients who were already known to have extensive disease.

A total of 11 other patients were studied in order to document the range of usefulness of this particular antibody in localising various tumour types. There appeared to be enough cross reaction with breast cancer to be useful but no clearly positive scans were obtained from patients with testicular neoplasms.

### Discussion

Monoclonal antibodies are promising potentially specific reagents for use in the diagnosis and therapy of cancer. We have shown, using a computerised subtraction technique, the ability of one $^{131}I$ labelled monoclonal antibody to detect metastiatic disease in patients with colorectal carcinoma. The limit of the resolving power of such an antibody is determined by the ratio of the antibody distributing in the tumour to that in the blood and adjacent tissue. This is influenced by shed antigen concentration in the blood as well as normal tissue cross reactions. For YPC2/12.1 there is little cross reaction except with polymorphonuclear leucocytes in the peripheral blood and with cells of the granulocyte series in the bone marrow. Despite this, localisation of metastases was successful using subtraction procedures to eliminate the image of blood borne antibody. The inherent physical limitations of this technique are compounded by both the poor counting statistics and by the activity of the $^{131}I$ in the $^{99m}Tc$ photon peak. More precise definition without subtraction may be obtained by use of computerised emission tomography but this requires complex equipment not widely available in general hospitals. While subtraction procedures are feasible for tumour localisation and can compensate for some of the unwanted tissue localisation of the labelled antibody, such undesired localisation could not be tolerated if monoclonal antibodies are to be used to deliver drugs or toxins for therapy. We are currently searching for suitable monoclonal antibodies for this purpose. In addition a prospective study using YPC2/12.1 is being undertaken in patients with colorectal carcinoma prior to definitive surgery to assess the efficacy of this technique compared with CT scanning for pre-operative staging.

We would like to thank our colleagues in the Department of Radiotherapy, Addenbrooke's Hospital, for referring patients for this study; Dr. A. Dixon for CT scanning; Mr. A.D. Lowe, Mrs. L. Croft and Mr. John Ellis for their expert technical help, and Mr. Charles Sampson for helpful advice.

### References

DYKES, P.W., HINE, K.R. BRADWELL, A.R. & 4 others. (1980). Localisation of tumour deposits by external scanning after injection of radiolabelled anti-carcinoembryonic antigen. *Br. Med. J.*, **i**, 220.

EPENETOS, A.A., NIMMON, C.C., MATHER, S., HAWKINS, L.R., GRANOWSKA, M. & BRITTON, K.E. (1982). Radioimmunoscintigraphy with $^{123}I$ monoclonal antibody. *Nucl. Med. Comm.*, **3**, 123.

FINAN, P.J., GRANT, R.M., MATTOS, C.D. & 4 others. (1982). The use of immunohistochemical techniques as an aid in the early screening of monoclonal antibodies. *Br. J. Cancer*, **46**, 9.

GALFRÉ, G., MILSTEIN, C. & WRIGHT, B. (1979). Rat × rat hybrid myelomas and a monoclonal anti-Fc portion of mouse IgG. *Nature* **277**, 131.

GOLDENBERG, D.M., KIM, E.E., DELAND, F.H., BENNETT, S. & PRIMUS, F.J. (1980). Radioimmunodetection of cancer with radioactive antibodies to carcinoembryonic antigen. *Cancer Res.*, **40**, 2984.

HALES, A. (1977). A procedure for the fusion of cells in suspension by means of polethylene glycol. *Somat. Cell Genet.*, **3**, 227.

LENNOX, E.S. & SIKORA, K. (1982). Definition of human tumour antigens. In : *Monoclonal Antibodies in Clinical Medicine*. (Eds. McMichael & Fabre). London: Academic Press, p. 111.

LEVINE, G., BALLOU, B., REILAND, J., SOLTER, D., GUMERMAN, L. & HAKARA, T. (1980). Localisation of $^{131}I$ labelled tumour specific monoclonal antibody in the tumour bearing Balb/c mouse. *J. Nucl. Med.*, **21**, 570.

MACH, J-P., CARREL, S., FORNI, M., RITSCHARD, J., DONATH, A. & ALBERTO, P. (1980). Tumour localisation of radiolabelled antibodies against carcinoembryonic antigens in patients with carcinoma. *N. Engl. J. Med.*, **5**, 310.

MACH, J-P., BUCHEGGER, F., GIRARDET, C. & 7 others. (1981). Uses of radiolabelled monoclonal anti CEA antibodies for the detection of human carcinomas by external photoscanning and tomoscintigra0hy. *Immunol. Today*, **2**, 239.

MILLER, R.A. & RUDDLE, F.H. (1976). Pluripotent teratocarcinoma thymus somatic cell hybrids. *Cell*, **9**, 45.

MOSHAKIS, V., MCILHINNEY, R.A.J., RAGHAVAN, D. & NEVILLE, A. M. (1981). Monoclonal antibodies to detect human tumours: an experimental approach. *J. Clin. Pathol.*, **34**, 314.

REPORT OF THE ADVISORY COMMITTEE ON CANCER REGISTRATION. *Cancer Registration in the 1980's*. London: HMSO.